# Residual Renal Function – How Fast Does the Residual Urine Output Function Decline in the First Year of Haemodialysis? – A Scoping Review

**DOI:** 10.3389/fneph.2021.808909

**Published:** 2022-01-26

**Authors:** Ulrich Steinwandel, Homa Kheirkhah, Hugh Davies

**Affiliations:** School of Nursing and Midwifery, Edith Cowan University, Joondalup, WA, Australia

**Keywords:** renal residual function, chronic kidney disease, haemodialysis, quality of life, fluid assessment

## Abstract

**Background:**

Haemodialysis is the most common treatment method in Australia for individuals requiring renal replacement therapy. Although it is known that the residual renal function in these patients has many advantages for their overall health outcomes and that the residual urine volume production is also declining over time, it is unknown how fast this functional decline occurs when patients are embarking on their first year on haemodialysis.

**Aim:**

This scoping review sought to determine if the functional decline in renal residual function in the first year of haemodialysis has been previously investigated, documented or quantified.

**Method:**

The scoping review was performed using variety of nursing and medical databases comprising MEDLINE, Embase, Web of Science and CINAHL Plus with Full Text.

**Results:**

The decline of renal residual function in patients on Peritoneal dialysis over the first year of treatment has previously been described, but not in detail for patients receiving haemodialysis. There is a paucity of knowledge how fast residual urine production can decline in patients receiving haemodialysis during their first year of treatment. A PRISMA checklist has been used to validate the results of this scoping review.

**Conclusions:**

The extended preservation of renal residual function in patients on haemodialysis is crucial for their survival and may have a positive impact on their quality of life. An observational study is needed to examine how fast the functional decrease of the residual urine production function within patients receiving haemodialysis generally occurs. This information could prove to be useful in the context of treatment goals and could inform clinical practice.

## Introduction

Chronic kidney disease (CKD) is a condition where the function of kidneys is diminished. In 2015, the global burden of the disease (GBD) attributed 1.2 million deaths to kidney failure. The GBD also estimated a further 1.2 million deaths and 18 million years of life lost from cardiovascular conditions directly related to a reduced glomerular filtration rate (GFR) in the kidneys (1). According to the Australian Bureau of Statistics (2), in 2017-2018 237,800 people in Australia (1% of the total population) were suffering from CKD. The prevalence of this disease remained unchanged with gender but increased with age (2). In 2017, one in nine deaths were attributed to CKD being an underlying and or an associated cause according to the Australian Institute of Health and Welfare (3). In 2016-17, about 16% of all hospitalisations were associated with CKD (3). Kidney and urinary diseases accounted for 1.4% of all diseases in Australia in 2015 (3). Indigenous Australians are 3.6% more likely to die from CKD than those of non-indigenous descent (3).

Individuals with chronic kidney disease (CKD) require a form of renal replacement therapy (RRT), either haemodialysis (HD), peritoneal dialysis (PD) or a kidney transplant, when they reach stage five of CKD (4). Traditionally, patients will start with RRT when the glomerular filtration rate (GFR) drops below 15mL/min/1.73m^2^ and uremic symptoms of kidney disease prevail (5). Haemodialysis sessions usually aim to restore fluid balance by removing excess fluid *via* ultrafiltration (UF) and remove metabolic waste products through a convective solute transport (6).

Residual renal function (RRF) in patients receiving dialysis is the residual ability of the kidneys to produce urine and excrete waste products, even when CKD persists. The importance of the RRF for patients on maintenance HD for their survival has often been emphasized (7–10). In addition to improved survival rates, a positive impact on their quality of life has also been reported, when RRF was present (11, 12). Further to this, the loss of RRF has also been associated with vascular calcification in HD patients (13), which can also have detrimental consequences on their health (14). Additionally, events of intradialytic hypotension (IDH) occurring while individuals undergo HD sessions have been described as being detrimental to their RRF (15).

Understanding of the RRF status of an individual is not just important for the extra clearance of harmful metabolites that maybe necessary during regular HD sessions (16), but it may also hold essential information about the daily urine volume. This in turn may help to inform fluid-related decision-making processes from patients and clinicians alike. An individualised, patient centred approach for the definition of treatment goals, particularly in regard to UF and excess volume, accounting for unique patient factors may prove to be beneficial for patient outcomes (17). While the knowledge of an individual’s current RRF has the potential to influence treatment regimen and treatment (UF) goals over time, it may also affect an individuals’ daily fluid intake in between HD sessions and affect their health-related quality of life (HRQL) and interdialytic weight gain (18–20). Knowledge of an individual’s RRF could potentially add significant information for clinicians and patients to lessen disease burden and improve disease management.

Although several studies have described the functional decline of the residual renal function in patients using PD (21, 22), there is a paucity of knowledge of the rate of the same functional decline in HD patients during their first year of treatment. With HD being the most common renal replacement therapy (RRT) modality in Australia (78% in year 2018) (23), this underscores the significance of the potential problem. The ability to quantify functional changes in RRF during the first year of HD, and in particular the residual urine volume (RUV) production ability holds the prospect of informing clinical practice by assisting clinicians to make clinical decisions on regular treatment goals including rate of ultrafiltration (UF) and total ultrafiltration goal (volume) prior to each HD session. So far, previous research has only described that RRF is declining in the first year of HD (24), and little evidence has been provided on how much the RRF is declining and how much this might vary amongst individuals over time.

## Aim

This scoping review aims to provide evidence in peer-reviewed published literature for the rate of functional decline in the urine output of CKD patients starting with maintenance haemodialysis during the first 12 months of treatment. This information could be useful to inform current clinical practice when patients embark on haemodialysis as their ongoing treatment for CKD. It could also potentially lead clinicians to implement novel structural measures to protect their patients’ RRF from functional decline in future. The following research question in this scoping review is: “What is the functional decline in urine production within the first 12 months following commencement of HD for end-stage renal disease (ESRD) and how does this affect their quality of life or mortality?”

To facilitate the literature search, the clinical question was addressed using the PICO framework with the following items:

Population: Adult CKD patients with ESRD, aged 18 years and above on maintenance HD in hospitals and satellite dialysis clinics were included, patients on home HD and pregnant patients were excluded from the search, Intervention: HD, Comparison: Residual Renal Output based on residual urine output volume compared to patients with no residual urine output, Outcomes: Quality of life, mortality.

## Methods

A scoping literature review was performed in 2021 to search for peer-reviewed journal articles in medical and nursing journals. The search mainly focused on residual renal function of patients within the first 12 months after haemodialysis initiation with emphasis on the residual urine output volume. It was of particular interest if residual urine output function provided a specific survival advantage in comparison to individuals with ESRD and no residual urine output. The databases used were CINHAL Plus with Full Text, MEDLINE, Embase and Web of Science. The search terms used included “residual renal function” or “residual kidney function” (= Search term 1 or S1) “haemodialysis initiation” (S2), “haemodialysis and urine output or urine volume” (S3), and “residual renal function and haemodialysis” (S3). The search terms S1, S2, S3 were combined with the term “first year of haemodialysis”. Journal articles included in this scoping review were peer-reviewed, full text articles, in English language and published between 2014 and 2021. Additionally, academic journals, renal registries, case reports, non-randomized clinical trials, doctoral dissertations and retrospective audits were included in the overall search. Further, articles and reviews which did not meet minimum quality requirements, without peer review or an abstract were excluded from the review. A PRISMA checklist has been applied to validate the results of this scoping review.

## Results

**Figure 1:**
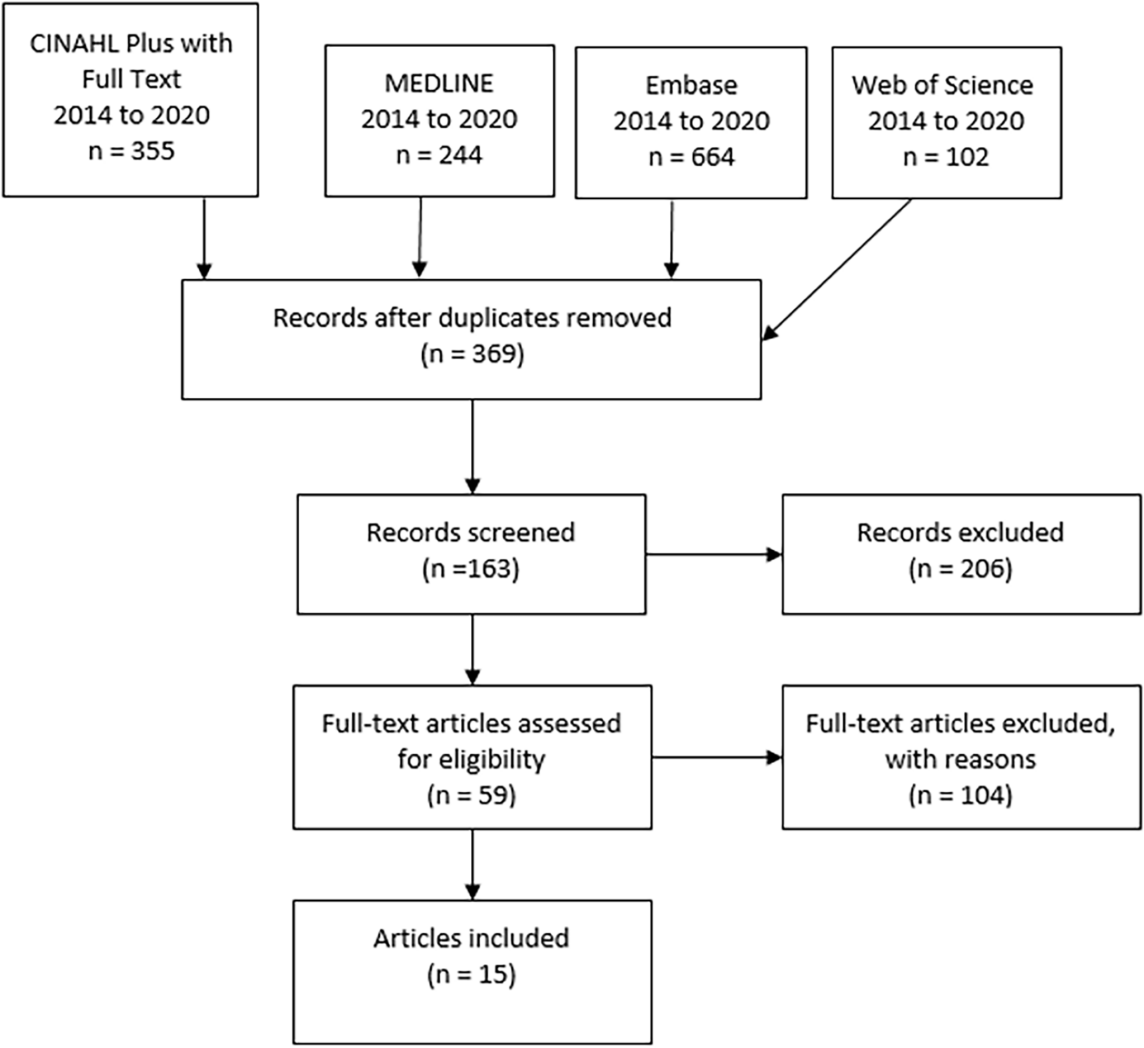
Results of scoping review by searching 4 databases (CINAHL plus, MEDLINE, Embase, Web of Science, PRISMA flowchart.

The primary search in all four databases resulted in 369 non-duplicate citations which were then screened (**Figure 1**). Applying inclusion and exclusion criteria reduced the number of qualified articles to 59. Another 104 articles were excluded during data extraction, as they did not contain enough statistical data or were as well not considered to be relevant to the research question of residual urine output during the first year of haemodialysis. This scoping literature review resulted in a definitive number of 9 articles which met all characteristics of the inclusion criteria. **Table 1** presents a summary of these 15 articles.

**Table 1:** Summary table of 9 articles: Decline of RRF and urine output volume during the first year of HD.

	Author (year), Title	Study design	Sample size and sites	Comments/key findings
1	Blum, D. (25) “Thinking Volume First: Developing a Multifaceted systematic approach to Volume Management in Haemodialysis.”	Case study presentation	16 patients HD unit St Michael’s Hospital (Canada)	Local volume management with 3 components Volume metric reporting integrated into the routine bloodwork reports. Abnormal metrics were evaluated using technological testing in the form of lung ultrasound and bioimpedance spectrometry. Patients with abnormal metrics for volume were reviewed and educated by the multidisciplinary team.
2	Wang, J. et al. (26) “A fast decline of residual renal function in the first year is a predictor for early withdrawal from peritoneal Dialysis in Non-diabetic patients.”	Observational study	567 patients at First Affiliated Hospital Zhejiang (China) who started with PD between 2005 and 2013	Faster RRF decline in the first year was a predictor for all-cause mortality and conversion to HD in non-diabetic PD patients
3	Lee et al. (27) “Ultrafiltration rate effects declines in residual kidney function in hemodialysis patients.”	Retrospective cohort study	7, 753 patients who initiated conventional hemodialysis from 2007 to 2011 (United States)	Higher UFR was associated with a rapid decline in residual renal function among conventional hemodialysis patients.
4	Obi et al. (9) “Residual kidney function decline and mortality in incident hemodialysis patients.”	Longitudinal study	6538 patients (2007 to 2010) (California, United States)	Only a few studies have explored RKF in patients on HD. RKF contributes to adequate solute clearance. Data at baseline and 1 year after HD initiation reported gradient association between RKF loss and all-cause mortality. Clinical benefits of RKF preservation methods are yet to be determined.
5	Shafi et al. (8) “Residual kidney function: Implications in the era of personalised medicine.”	Case study	(Amsterdam, Netherlands)	Residual kidney function is essential for improved outcomes of PD and HD. After dialysis initiation, RKF provides volume and solute clearance necessary to remove toxins that cannot be removed using conventional dialysis methods. The management of uremia can be personalized due to the benefits of RKF, taking into account the solute, volume and quality of life needs.
6	Silva et al. (20) “Coping with fluid restriction and the quality of life in hemodialysis patients with very low or no daily urine output.”	Cross sectional study	271 Brazilian adult maintenance hemodialysis patients (Brazil)	52.4% of the participants reported being moderately to extremely bothered by fluid restriction and had lower scores for health-related quality of life. Increased fluid restriction decreases quality of life. An improvement in fluid restriction methods can improve quality of life in HD patients.
7	Sjolund et al. (28) “Diuretics, limited ultrafiltration, and residual renal function in incident hemodialysis patients: A case series.”	Case series	7 hemodialysis patients (The Netherlands)	During the first 6 to 12 months the mean urine volume was reduced by 1ml per month, residual GFR declined by 0.03 ml/min/1.72m^2^ /month. The mean rate of decline for urine volume from 12 to 24 months was 33ml/month and residual GFR was 0.02ml/min/1.72m^2^ /month. The low rate decline is due to the effects of diuretic (frusemide) during the first 2 years of HD initiation.
8	Poulsen et al. (29) “Quality of life development during initial haemodialysis therapy and association with loss of residual renal function.”	Questionnaire (Kidney Disease Quality of Life Short Form Version 1.3)	82 patients at 6 months and 12 months of dialysis treatment (Denmark)	The use of Irbesartan did not affect Health Related Quality of Life (HRQL). Decline in GFR correlated with decreased HRQL over time. Preservation of residual renal function is important for HRQL, which is also negatively affected by comorbidities such as diabetes, hospital admissions, female gender and age.
9	Mokoli et al. (30) “Factors associated with residual urine volume preservation in patients undergoing haemodialysis for end-stage kidney disease in Kinshasa.”	Cohort study	250 patients with ESRD undergoing haemodialysis between January 2007 and July 2013 in two haemodialysis centres in Kinshasa, Democratic Republic of Congo	ACE inhibitors, chronic tubulointerstitial nephropathy and left ventricular hypertrophy reduction emerged as the main independent predictors of residual urine volume preservation over time, and in particular in the first year of dialysis.

6 articles were found covering this topic which were either Systematic Literature reviews or Systematic Literature reviews. These articles are presented in **Table 2**.

**Table 2:** Summary table of 6 articles, either Literature Reviews or Systematic Literature reviews: Decline of RRF and urine output volume during the first year of HD.

	Author (year), Title	Study design	Sample size and sites	comments/key findings
1	Huang, S. et al. (31) “Euvolemia in haemodialysis patients: A potentially dangerous goal?”	Literature review	12 articles (1999 to 2016) (United Kingdom)	Patient characteristics such as RRF, adequate volume control, lack of significant anaemia/electrolyte imbalance, satisfactory health related quality of life, low comorbid disease burden, and good nutritional status. Regular RRF measurements is essential, because HD frequency needs to be adjusted depending on the patient’s RRF status.
2	Kong, Davies, & Mount (24) “The importance of residual kidney function in dialysis patients.”	Systematic literature review	Articles from 2017 to 2018 (Victoria, Australia)	Over 80% of patients have some levels of residual renal function present at the start of dialysis treatments. There is a decline over the years, however, up to 30% of patients after 5 years of HD still have a measurable level of RRF. In HD patients, RRF is usually disregarded. To maintain and maximize RRF in HD patients, further research is needed.
3	Mathew, A.T. (17) “Incremental dialysis for preserving residual kidney function: Does one size fit all when initiating dialysis?”	Literature review	12 observational cohort studies (California, United States)	Haemodialysis is usually initiated on 3 times per week, regardless of residual kidney function. As RKF is associated with numerous benefits for the patients, it is essential for HD treatments to be personalized, taking into account the individual RKF. Incremental HD prescriptions are recommended in this case, as HD initiation is less frequent and allows for regular measurement of RKF.
4	OK et al. (32) “Interplay of volume, blood pressure, organ ischemia, residual renal function, and diet: Certainties and uncertainties with dialytic management.”	Literature review	(Turkey)	Both blood pressure and volume control are managed poorly in HD patients which causes the increased morbidity and mortality. Restriction of dietary salt intake, increased frequency, and/or duration of haemodialysis sessions or addition of temporary extra sessions during the process of gradually reducing post- dialysis body weight in conventional HD and discontinuation of antihypertensive medications could prevent these complications.
5	Mitema & Jaar (33) “How can we improve the quality of life of dialysis patients.”	Literature review: review of various Health-Related Quality of Life (HRQOL) tools	(United States)	There are multiple validated tools which can be used to improve HRQOL in ESRD patients. Each tool should be used in an individualized manner to address specific dialysis patient needs such as anaemia, depression sexual dysfunction, sleep related disorders and the preservation of residual kidney function.
6	Fang et al. (34) “Residual renal function among patients on haemodialysis and implications for clinical practice.”	Systematic Literature review	4 databases searched for relevant information between 200 and 2018 (Medline, CINAHL Plus, Embase and Pubmed)	Nephrology nurses can improve clinical practice to assist preserving RKF in HD patients through preventing intradialytic hypotension, improving dry weight assessment and volume control, advocating for incremental HD, promoting haemodiafiltration and other measures

## Residual Renal Function During the First Year of Haemodialysis

Relevant literature concerning urine volume output of patients receiving maintenance HD during their first year on RRT was examined. No articles quantifying HD patients’ declining urine volume during this time period were found, however, several papers have concluded that the RRF rapidly declines during the first year of maintenance HD. There was no further detailed description of this declining RRF found in the current scientific literature or describing specifically the RUV in this particular patient cohort.

An observational study by (26) has concluded that a fast decline in RRF in PD patients resulted in early withdrawal from PD and conversion to HD treatment, as HD allows for more precise excess volume management. On the other hand, large volume removal (UF goals) during HD may have negative consequences, such as IDH and subsequently a decline in RRF (31). These authors concluded that frequent RRF measurements are essential, as the frequency of HD treatments need to be adjusted according to the patient’s RRF status.

A small observational study on seven HD patients by Sjolund, Garcia Anton (28) in the Netherlands has reported that during the first six to 12 months of maintenance HD the mean urine volume reduced at a rate of 1 ml per month. These authors emphasized the beneficial effects of loop diuretics on RRF as their patients were concurrently treated with increasing doses of furosemide during the first 12 months of HD treatment and suggested their use in all incident HD patients. According to their conclusion, this seems to be not a commonly used approach and may vary amongst HD centres globally.

A prospective multicentre study in the United States by You, Kalantar-Zadeh (35) has concluded, that a large proportion of patients commencing with HD have a substantial residual kidney function including urine output and that “some data suggests that haemodialysis patients experience greater preservation of kidney function than previously estimated”. These authors have also reported that sometimes 14-20% of patients still have some RRF after three to five years after transitioning to HD.

Mokoli et al. (30) conducted a historical cohort study, recruiting 250 patients with end stage renal disease (ESRD). The study collected residual urine volume (RUV) over 24 hours at the start, 6 months and 12 months of HD treatments. Patients who had RUV of > 500 ml/day were mostly on diuretics, angiotensin converting enzyme inhibitors (ACEI) with less hypovolemia, hyperkalaemia and anaemia, with higher levels of serum albumin (30), which could prove a survival benefit and affect mortality. The mean RUV values at HD initiation were 680 ± 537 ml/day, 558 ± 442 at 6 months and 499 ± 475 at 12 months. Decreased urine volume levels were seen in all patients except in patients who did not have any hypertension. Patients who were male, hypovolemic, had chronic tubulointerstitial nephropathy and those who were treated with diuretics and ACEI and angiotensin receptor blockers (ARB) had a higher median urine volume (36). The study concluded that RRF declines over 12 months of HD treatments. Preservation of RRF was significantly associated with ACEI and interstitial nephropathy. On the other hand, the loss of RRF was associated with the presence of left ventricular hypertrophy (LVH) at the initiation of HD treatments (36). The main limitation in this study was the relatively small sample size and the retrospective characteristics that hindered setting up a cause-effect relationship. Despite the limitations, this study is one of the few that has used RUV collections to measure the decline in RRF over 12 months of HD treatments. The incorporation of the RRF into the HD prescriptions has potential to aid in preserving residual kidney function in the long term. Most clinics do not measure RRF due to the difficulty of timed urine collection and therefore the patient does not receive personalised HD prescription that may aid in the preservation of RRF (37). Wong et al. (37) explored methods of measuring RRF without urine collection. The study concluded that timed urine collections can be very challenging and instead the use of biomarkers to determine cut off levels of residual urea clearance may be a better and promising method. You, Kalantar-Zadeh (35) have reported correspondingly that frequently measuring 24-hour urine collection is onerous and assessment of patient-reported urine volume may help in the day-to day management of HD and can be easily implemented at the patient’s bedside.

## Quality of Life and Residual Renal Function

Another major factor in the discussion about RRF is its impact on the Quality of Life (QOL) in HD patients. A publication by Mitema and Jaar (33) has investigated a variety of validated tools which are readily available to assess HD patients QOL. They have recommended to use each tool in an individualized manner to address specific patient needs and recommended to include the preservation of the RRF. These authors also reported from patients with self-reported urine output at baseline of at least 250 ml/day were reporting better social functioning, vitality, cognitive functioning and quality of life over a year. Poulsen et al. (18) surveyed 82 HD patients using the Kidney Disease Quality of Life Short Form Version 1.3 (KDQOL-SFTM) at the start of treatment, at six and 12 months. The study concluded that health related quality of life (HRQOL) is largely impacted in patients receiving HD treatment. Although the patient can become used to the HD and this could lead to an improved HRQOL. However, other debilitating factors such as multiple side effects of HD, frequent hospitalisation, diabetes and the decline in RRF after 12 months can eventually lead to a further decrease in HRQOL (18). Decreasing glomerular filtration rate (GFR) was measured by collecting urine for 24 hours at baseline, 6 and 12 months in this study. A decreasing GFR in patients with CKD also lowers HRQOL, was concluded by these authors. Other studies have found a marked correlation between decreased urine volume and a decreased quality of life. The lower HRQOL is associated with increased restrictions in diet, fluids, and the duration of dialysis sessions as the RRF declines (18, 20). Kong, Davies and Mount (24) conducted a literature review to explore the relationship between RRF and the outcomes for patients on HD treatment. The study found that the preservation of RRF, even at very low levels, contributes to increased quality of life and patient survival (24).

## Residual Renal Function Preservation

In a longitudinal cohort of 6538 patients, Obi et al. (9) aimed to determine the existing clinical factors at HD initiation that predict the preservation of RRF at 1 year of HD treatments. The study also included quantitative investigations of the association in the annual change of RRF with survival. The study found that greater renal urine clearance (CLurea) after 1 year of HD treatments, which lead to better survival rates. Factors such as being female, non-white races, history of congestive heart failure (CHF) and diabetes were the main factors which were associated with RRF decline in the first year of HD (9). The median baseline urine volume was 900 ml/day and the median volume at 1 year was 650 ml/day. These authors also observed a significant trend toward lower mortality at higher urine volume. Several limitations existed in this study with the most important one being the inaccuracy of RRF measurements, using the (CLurea) instead of the average renal urea and creatinine clearance. Complete urine collection samples were also challenging in terms of timing. Patients who were treated with PD, nocturnal HD or Home HD were excluded from the study although they might have had presented with different RRF (9).

In HD patients, RRF can contribute to removal of sodium and improved volume control, leading to a reduced interdialytic weight gain. Left ventricular hypertrophy and left ventricular systolic dysfunction are less severe with the presence of RRF. Residual renal function is also associated with higher serum albumin and correlates to a better nutritional status in HD patients (24, 38). Mathew et al. (17) have emphasized the importance of regular RRF measurements in order to allow the use of incremental HD in case of a decline in RRF. The difficulty in accurate measurement of the interdialytic urine levels is thought to be the main reason that only 5% of HD patients have measured RRF levels. Higher RRF at one year of dialysis has been associated with higher survival rates (17). Studies have suggested the preservation of RRF may be possible with a twice weekly HD regimen, compared to the routine thrice weekly which was associated with a loss of RRF that was seven times higher (17, 39).

A systematic literature review by Fang, Lunardi (34) has indicated that renal nurses have multiple options to improve clinical practice which may assist in preserving RRF in HD patients through a variety of measures. This includes the prevention of intradialytic hypotension, improving measures for dry weight or ideal body weight assessments, volume control, advocating for incremental HD, promoting haemodiafiltration (HDF) as a treatment option and other measures.

## Discussion

In the HD population, volume overload and hypovolemia are common debilitating symptoms which can increase hospitalisation, leading to a reduced quality of life (25). Volume overload can cause cardiovascular events, dyspnoea and left ventricular remodelling which can eventually lead to death (40). Hypovolemia on the other hand causes organ ischemia, fatigue and changes in cognitive functioning (25, 32) while organ ischemia may subsequently lead to loss of residual renal function (32). Blum et al. (25) suggested three main volume management strategies that will help clinicians determine patient’s true volume status. The first strategy was to integrate the reporting of volume metric into the routine bloodwork report. The use of technology such as bioimpedance and lung ultrasound along with traditional tests can aid in volume assessment and review and assessment of abnormal volume metrics by the multidisciplinary team. The study identified 16 patients with abnormal volume metrics where the volume dysfunction was related to either a high intradialytic weight gain, incorrect target weight gain or missed sessions of HD treatment (25). The patients were tracked at 6-week intervals with the goal of reducing the prevalence of abnormal volume metrics by targeting the root causes. The study concluded that the use of the three volume management strategies can personalise HD treatments and improve volume status in patients (25). The study had several limitations including the tracking of the patients in 6-week intervals and not in real time and the use of recommended technological adjuncts being limited to the availability of trained staff (25). A recent multi-centre randomized controlled trial study by (41) has demonstrated the efficacy of a lung ultrasound-guided treatment strategy when assessing patients for fluid overload and determining ultrafiltration goals. This highlights the need for qualified and appropriately trained healthcare professionals, which potentially will be in most situations the renal nurses who are caring for HD patients attending their treatments.

HD has often been described as being associated with being more successful in attaining euvolemia than PD, but also with a more rapid loss of residual renal function (42) which may be detrimental for the patient’s survival (8).

A study by Lee, Okuda (27) has indicated that a higher ultrafiltration rate (UFR), which equals the ultrafiltration in millilitres per hour of HD treatment, has been observed as associated with rapid decline in RRF and also increased mortality among conventional HD patients. It can therefore be assumed that the lower the UFR is, the less detrimental effect the UFR may have on the RUV in the long term.

## Implications for Practice

- Renal nurses are the primary healthcare professionals caring for patients starting on maintenance HD and must be made aware and receive more specific education about the importance of maintaining RRF and RUV in HD patients, specifically during the first year on HD
- Regular RUV measurements in patients starting with HD may hold essential information to inform clinicians when deciding on regular treatment (UF) goals
- Knowledge of the individual parameters of RUV may have positive implications for HRQOL and provide better health outcomes for HD patients starting with HD
- Increasing doses of loop diuretics during the first year of HD, such as furosemide, may be preservative for the RRF and RUV.
- Observing of patient-reported urine volume may provide for better fluid management and may support preserving RRF and RUV in patients on HD.

## Conclusion

This literature review has demonstrated, that preserving RRF and RUV for as long as possible has multiple positive implications on the health-related quality of life (HRQOL) and on the morbidity in HD patients. This is essential and important, especially the longer this RRF is maintained. No study was found which had either observed, documented, or quantified the loss of RUV in a detailed description over the first 12 months of maintenance HD and/or any correlation to goals of treatment in terms of removal of excess fluid (UF). This critical information could potentially be very useful and has potential to inform clinical practice on an individual patient level. Future research should be undertaken to observe this common change in RRF including urine production during the early stages of maintenance HD, to provide essential knowledge to clinicians and patients alike. This information could have potential to impact on HRQOL and health outcomes for HD patients.

## Limitations and Future Studies

Renal nurses who are caring for patients starting on maintenance haemodialysis must be made aware and receive more education about the importance of maintaining renal residual function in patients receiving haemodialysis, specifically during the patient’s first year on haemodialysis. This may affect health outcomes for this patient cohort positively and may have potential to result in a prolonged renal residual function for these patients.

The following table (**Table 3**) depicts potential future studies resulting from this scoping review.

**Table 3:** Potential future studies resulting from this scoping review.

An observational study on the functional decrease of the renal residual function including urine production in haemodialysis patients during their first year of treatment	Study 1
An observational study on the current clinical practices and methods of fluid assessment and ultrafiltration goals in haemodialysis units	Study 2
What is the current clinical practice of renal nurses in terms of preserving the renal residual function in patients on haemodialysis – An observational study	Study 3
Which factors may cause the decline of the renal residual function and the urine production in haemodialysis patients?	Study 4

## Data Availability Statement

The original contributions presented in the study are included in the article/supplementary material. Further inquiries can be directed to the corresponding author.

## Author Contributions

US and HK conducted the systematic literature review, performed initial data analysis, selected papers and appraised their quality, read and approved the final manuscript. HD participated in the systematic review, confirmed the analysis and helped to draft the manuscript, read and approved the final manuscript. All authors contributed to the article and approved the submitted version.

## Conflict of Interest

The authors declare that the research was conducted in the absence of any commercial or financial relationships that could be construed as a potential conflict of interest.

## Publisher’s Note

All claims expressed in this article are solely those of the authors and do not necessarily represent those of their affiliated organizations, or those of the publisher, the editors and the reviewers. Any product that may be evaluated in this article, or claim that may be made by its manufacturer, is not guaranteed or endorsed by the publisher.
